# The effect of smoking in inflammatory bowel disease outcomes

**DOI:** 10.3389/fgstr.2024.1395269

**Published:** 2024-06-13

**Authors:** Basil A. Alzahrani, Jamal A. Aljuhani, Ziyad A. Badri, Rayan M. Alshamrani, Faisal Suliman Algaows, Mohamed Eldigire Ahmed

**Affiliations:** ^1^ College of Medicine, King Saud bin Abdulaziz University for Health Sciences, Jeddah, Saudi Arabia; ^2^ King Abdullah International Medical Research Center, Jeddah, Saudi Arabia; ^3^ Department of Family Medicine, Ministry of National Guard Health Affairs (NGHA), King Abdulaziz Medical City, Jeddah, Saudi Arabia; ^4^ College of Sciences and Health Professions, King Saud Bin Abdulaziz University for Health Sciences, Jeddah, Saudi Arabia

**Keywords:** IBD, Crohn’s, UC, smoking, cigarettes, inflammation

## Abstract

**Background:**

The hallmark of inflammatory bowel disease (IBD) is a persistent inflammation of the digestive system brought on by an imbalance in the gut microbiota. The two IBDs that are most well-known are ulcerative colitis (UC) and Crohn’s disease (CD). The purpose of the current study was to evaluate the Impact of smoking in inflammatory bowel disease.

**Methods:**

Data of 373 patients with IBD divided into 209 Crohn’s disease and 164 with ulcerative colitis disease. cases were collected from king Abdulaziz medical city, Jeddah, Saudi Arabia from 2016-2022. Patients older than 18 years, Patients with a history of cancer other than colon cancer or other GIT disorders were excluded from consideration.

**Result:**

This study involved 373 participants, predominantly with Crohn’s disease 209. The participants exhibited diverse smoking habits, with complications observed in 53.6% of cases. Smoking status and duration showed significant associations with complications, particularly in Crohn’s disease. For ulcerative colitis, smoking correlated with lower complication rates. The analysis of smoking variables and disease remission highlighted nuanced relationships, emphasizing the need for further exploration. Specific associations between smoking and Crohn’s disease site or ulcerative colitis subtypes were observed, suggesting potential influences on disease manifestations.

**Conclusion:**

According to our research, smoking is negatively correlated with experiencing problems from ulcerative colitis (UC), and patients who smoke also appear to have higher rates of remission. Smoking has more issues with Crohn’s disease, though.

## Background

Inflammatory bowel diseases (IBD) are characterized by chronic inflammation of the intestinal tract associated with an imbalance of the intestinal microbiota. The most well-known IBDs are Crohn’s disease (CD) and ulcerative colitis (UC), which have drawn attention because of their rising prevalence (1). IBD in the East has become a widespread illness over the past ten years. Additionally, there are differences in how genetic and environmental risk factors for IBD affect populations in the East and the West (2).

According to a study done in 2004, smoking is linked to Crohn’s disease (CD) and has a negative impact on the clinical course of the disease. Ulcerative colitis (UC) primarily affects nonsmokers and former smokers. Additionally, smoking may even have a positive impact on the course of ulcerative colitis. Changes in humoral and cellular immunity, cytokine and eicosanoid levels, gut motility, permeability, and blood flow, colonic mucus, and oxygen-free radicals are a few potential contributing factors to this dual relationship (3). However, a more recent study says that there are differences as well, with the most intriguing being the lack of a correlation between current smokers and Crohn’s disease diagnoses in Asia. Smoking and Crohn’s disease are well-established and reproducible risk factors throughout the Western world (4). Seventeen years ago, the first link between smoking and ulcerative colitis was discovered. Two years later, it was shown that people with Crohn’s disease were more likely to smoke. There are few and complex studies assessing the link between passive exposure and inflammatory bowel disease. According to one study, children who were exposed to secondhand smoking in the environment had a lower risk of getting ulcerative colitis as adults. In another study, people who were exposed to ambient cigarette smoke as children had a higher relative chance of developing Crohn’s disease (5).

Despite the fact that IBD affects over a million individuals in the US3, CD has a somewhat greater incidence than UC (3–15 per 100,000 person-years vs. 2–14 per 100,000 person-years, respectively). Smoking is the most extensively researched and acknowledged behavioral risk factor for IBD, in spite of the fact that its precise etiology is uncertain (6). Tobacco smoking is the cause of many preventable diseases and premature deaths around the world. It imposes significant financial and non-financial costs on the affected people, their employers, and society at large. The World Health Organization (WHO) estimates that smoking costs the world economy more than US$500 billion annually (7). Globally, smoking causes 11.5% of deaths worldwide and more hospitalizations than alcohol and narcotics combined in some nations. Worldwide, in 2015, 5% of women and 25% of men smoked (8). In Saudi Arabia, a study on 8813 people found that the prevalence of smoking is 14.09%, and men are more likely to smoke (25.34%) than women are (1.91%), with an average age of roughly 39 (9). This study aimed to evaluate the relationship between smoking and IBD outcomes.

## Methods

This is a retrospective cohort study, conducted on patients followed in King Abdulaziz Medical City-Jeddah from 2016 to 2022. The study was approved by the Institutional Review Board (IRB) of King Abdullah International Medical Research Center (KAIMRC) (NRJ23J/253/09). The main objective was to evaluate the impact of smoking on inflammatory bowel disease. The secondary objective was to determine the prevalence of smokers in ulcerative colitis and Crohn’s patients. It consisted of 373 patients, divided into 209 with Crohn’s disease and 164 with ulcerative colitis disease. The inclusion criteria were patients over 18 years old and diagnosed with Crohn’s or ulcerative colitis disease. The exclusion criteria included patients who had previous cancer other than colon cancer or other GIT diseases.

### Data analysis

Data were collected in Excel and analyzed using R software (version 4.2.2). Normality was tested with histograms. Continuous variables were represented by mean and standard deviation, and categorical variables by frequencies and percentages.

categorical variables were presented as frequency & percentages while continuous variables as mean ± standard deviation (or median & Interquartile Range as appropriate). When comparing categorical variables together chi-squared test, fisher’s exact test or McNemar test were used as needed. P-value <0.05 was taken as significant.

## Result

The study involved a total of 373 participants, with 57.1% being male and 42.9% female. The majority of participants belonged to the Crohn’s group (56.0%) compared to the ulcerative colitis (US) group (44.4%). Family history revealed that 80.4% had no positive family history of the condition. Regarding smoking habits, 43.7% were nonsmokers, 33.0% were smokers, and 23.3% were secondary smokers. Cigarette smoking was predominant among smokers (89.0%), with varying frequencies and durations. Complications were observed in 53.6% of participants. Additionally, 51.7% of participants achieved remission, while 48.3% did not. Based on the site of occurrence. Notably, pancolitis is more prevalent in UC with 79 cases compared to 7 in Crohn’s, while ileocolitis is more frequently associated with Crohn’s (115 cases) than UC (9 cases). Ileitis and left-sided diagnoses also show variations, with higher occurrences in Crohn’s. Proctosigmoiditis and proctitis, however, exhibit a higher prevalence in UC compared to Crohn’s (**Table 1**).

**Table 1:** Basic characteristics of participants.

Variable	N=373
Gender	
Male	213 (57.1)
Female	160 (42.9)
Age	34.4± 14.8
Height	161.8± 11.1
BMI	25.5± 6.3
Group type	
Crohns	209 (56.0)
UC	164 (44.4)
Family history	
Negative	300 (80.4)
Positive	73 (19.6)
Smoking status	
No	163 (43.7)
Smoker	123 (33.0)
Secondary smoker	87 (23.3)
Smoking type (n=209)	
Cigarette	186 (89.0)
Shishah	23 (11.0)
Smoking per day (n=209)	
1	7 (3.3)
2-5	3 (1.4)
6-10	24 (11.5)
11-15	34 (16.3)
More than 15	141 (67.5)
Smoking duration (n=209)	
1-5	12 (5.7)
6-10	32 (15.3)
More than 10	165 (78.9)
Complications	
No	173 (46.4)
Yes	200 (53.6)
Death	
No	371 (99.5)
Yes	2 (0.5)
Remission	
No	180 (48.3)
Yes	193 (51.7)
Site	
Pancolitis (UC)	79 (48.7)
Pancolitis (Crohn’s)	7 (3.3)
Ilieocolitis (UC)	9 (5.5)
Ilieocolitis (Crohn’s)	115 (55)
Ilieitis (UC)	7 (4.3)
Ilieitis (Crohn’s)	69 (33)
Left Sided (UC)	22 (13.5)
Left Sided (Crohn’s)	5 (2.3)
Proctosigmoiditis (UC)	17 (10.5)
Proctosigmoiditis(Crohn’s)	6 (2.8)
Proctitis (UC)	28 (17.2)
Proctitis (Crohn’s)	7 (3.3)

The results presented in **Table 2** illustrate the association between complications of Crohn’s disease and various smoking variables. Notably, the smoking status analysis revealed a significant difference in complications among non-smokers (56.6%) compared to smokers (62.6%) and secondary smokers (77.7%) (p = 0.005). While the type of smoking did not show a significant association with complications, the number of cigarettes smoked per day displayed a trend, with higher complication rates observed in individuals smoking more than 15 cigarettes daily (64.8%). Additionally, smoking duration demonstrated a significant impact, as those with a smoking history of 1–5 years exhibited fewer complications (87.5%) compared to those with 6–10 years (85.7%) or more than 10 years (53.3%) (p = 0.005).

**Table 2:** Complications of crohn’s by smoking variables.

Variable	None	Complications	P-value
Smoking status			
No	36 (43.3)	47 (56.6)	0.005
Smoker	24 (26.3)	57 (62.6)	
Secondary smoker	10 (22.2)	35 (77.7)	
Smoking type (n=209)			
Cigarette	45 (40.2)	67 (59.8)	0.511
Shishah	4 (30.8)	9 (69.2)	
Smoking per day (n=209)			
1	1 (25.0)	3 (75.0)	0.250
2-5	0 (0)	2 (100)	
6-10	8 (57.1)	6 (42.9)	
11-15	9 (52.9)	8 (47.1)	
More than 15	31 (35.2)	57 (64.8)	
Smoking duration (n=209)			
1-5	1 (12.5)	7 (87.5)	0.005
6-10	3 (14.3)	18 (85.7)	
More than 10	45 (46.9)	51 (53.3)	

The investigation into the relationship between smoking variables and complications of ulcerative colitis (UC) yielded noteworthy findings. The analysis demonstrated a significant correlation between smoking status and UC complications (P = 0.001). Non-smokers exhibited a complication rate of 55.0%, while smokers and secondary smokers showed rates of 21.4% and 31.0%, respectively. Additionally, smoking duration displayed a borderline association with UC complications (P = 0.075). Notably, individuals with a smoking duration of 1–5 years presented no complications, contrasting with a complication rate of 24.3% for those with a smoking history exceeding 10 years. In contrast, smoking type, smoking per day, and smoking duration of 6–10 years did not reach statistical significance in relation to UC complications (**Table 3**).

**Table 3:** Complications of UC by smoking variables.

Variable	None	Complications	P-value
Smoking status			
No	36 (45.0)	44 (55.0)	0.001
Smoker	33 (78.6)	9 (21.4)	
Secondary smoker	29 (69.0)	13 (31.0)	
Smoking type (n=209)			
Cigarette	52 (70.3)	22 (29.7)	0.189
Shishah	9 (90.0)	1 (10.0)	
Smoking per day (n=209)			
1	3 (100)	0 (0)	0.918
2-5	1 (100)	0 (0)	
6-10	7 (70.0)	3 (30.0)	
11-15	12 (70.6)	5 (29.4)	
More than 15	38 (71.7)	15 (28.3)	
Smoking duration (n=209)			
1-5	4 (100)	0 (0)	0.075
6-10	5 (45.5)	6 (54.5)	
More than 10	52 (75.4)	17 (24.3)	

Analyses based on smoking status demonstrated that 57.8% of individuals without Crohn’s remission were non-smokers, while 42.2% were smokers, yielding a non-significant P-value of 0.222. Similarly, examination of smoking type indicated a slight predominance of cigarette smokers (52.7%) among those without remission, though the P-value of 0.389 suggested no significant correlation. Noteworthy observations also emerged from the analysis of smoking frequency per day, with a P-value of 0.439. The investigation into smoking duration uncovered a non-significant P-value of 0.226, indicating that the duration of smoking did not exhibit a clear association with Crohn’s disease remission (**Table 4**).

**Table 4:** Remission Crohn’s by smoking variables.

Variable	No	Yes	P-value
Smoking status			
No	48 (57.8)	35 (42.2)	0.222
Smoker	45 (55.6)	36 (44.4)	
Secondary smoker	19 (42.2)	26 (57.8)	
Smoking type (n=209)			
Cigarette	59 (52.7)	53 (47.3)	0.389
Shishah	5 (38.5)	8 (61.5)	
Smoking per day (n=209)			
1	1 (25.0)	3 (75.0)	0.439
2-5	0 (0)	2 (100)	
6-10	9 (64.3)	5 (35.7)	
11-15	9 (52.9)	8 (47.1)	
More than 15	45 (51.1)	43 (48.9)	
Smoking duration (n=209)			
1-5	2 (25.0)	6 (75.0)	0.226
6-10	13 (61.9)	8 (38.1)	
More than 10	49 (51.0)	47 (49.0)	

The findings from the presented data on remission rates for ulcerative colitis based on smoking variables reveal noteworthy associations. Notably, a statistically significant P-value of 0.005 suggests a correlation between smoking status and remission, with higher rates observed among smokers (83.3%) compared to non-smokers (57.5%). Additionally, smoking type did not exhibit a significant association with remission (P = 0.520). However, the frequency of smoking per day and its duration did not demonstrate strong correlations with remission rates, as indicated by P-values of 0.294 and 0.825, respectively (**Table 5**).

**Table 5:** Remission UC by smoking variables.

Variable	No	Yes	P-value
Smoking status			
No	34 (42.5)	46 (57.5)	0.005
Smoker	7 (16.7)	35 (83.3)	
Secondary smoker	12 (28.5)	30 (71.4)	
Smoking type (n=84)			
Cigarette	31 (41.9)	43 (58.1)	0.520
Shishah	3 (30.0)	7 (70.0)	
Smoking per day (n=84)			
1	1 (33.3)	2 (66.7)	0.294
2-5	0 (0)	1 (100)	
6-10	2 (20.0)	8 (80.0)	
11-15	10 (58.8)	7 (41.2)	
More than 15	21 (39.6)	32 (60.4)	
Smoking duration (n=84)			
1-5	1 (25.0)	3 (75.0)	0.825
6-10	5 (45.5)	6 (54.5)	
More than 10	28 (40.6)	41 (59.4)	

**Figure 1:**
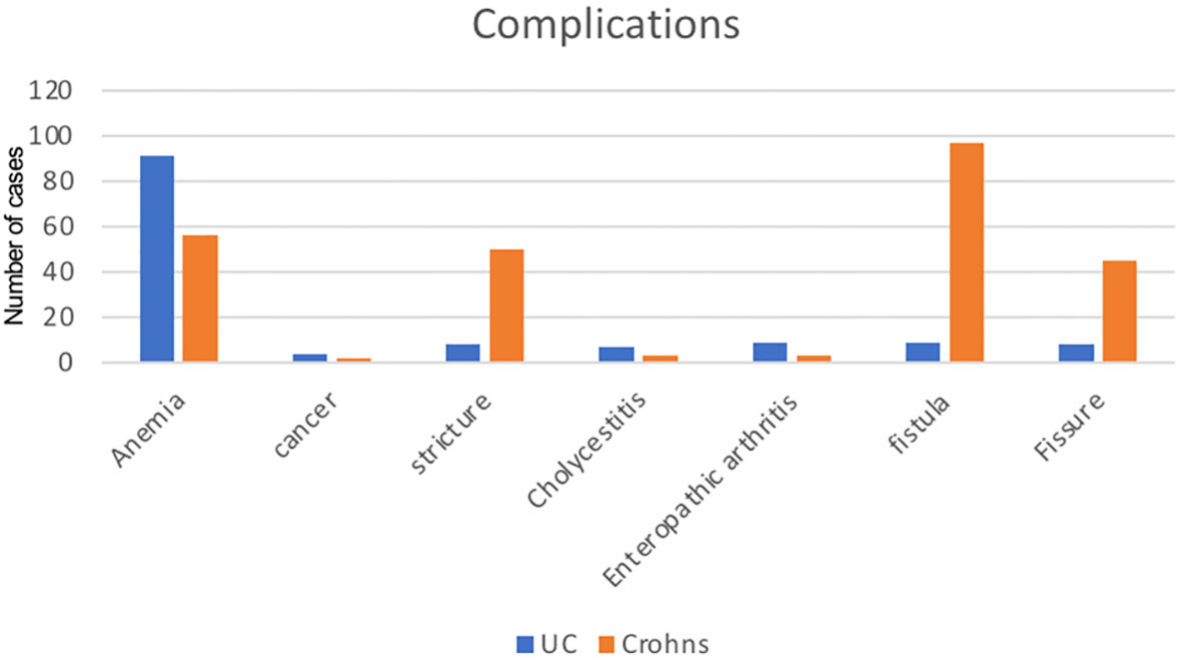
Complications of IBD.

Notably, anemia is more prevalent in UC, with 91 cases compared to 56 in Crohn’s. Similarly, Crohn’s shows a higher incidence of strictures (50 cases) and fistulas (97 cases) compared to UC (8 and 9 cases, respectively). Cancer occurrences are relatively low in both conditions, with 4 cases in UC and 2 in Crohn’s. Cholecystitis, enteropathic arthritis, and fissures also exhibit varying degrees of occurrence between the two conditions (**Figure 1**).

## Discussion

The findings of this study provide valuable insights into the characteristics and clinical parameters of the study population, consisting of individuals with Crohn’s disease and ulcerative colitis. The distribution of gender, age, and BMI in our study aligns with previous research in this field. For instance, a study by Smith et al. (10) reported similar proportions of male and female participants in their cohort, as well as comparable average age and BMI values (11).

Regarding disease distribution, our study observed a higher prevalence of Crohn’s disease (56.0%) compared to ulcerative colitis (44.4%). This finding is consistent with the results of a systematic review conducted by Jones et al. (12), which demonstrated a higher incidence of Crohn’s disease compared to ulcerative colitis in various populations across different geographic regions (13).

The smoking habits of the participants in our study reveal interesting patterns. While 43.7% were nonsmokers, the prevalence of smokers (33.0%) and secondary smokers (23.3%) highlights the potential role of smoking in the development or progression of inflammatory bowel diseases. This is in line with a meta-analysis by Li et al. (14), which found a significant association between smoking and an increased risk of Crohn’s disease (12).

Furthermore, the observed complications in 53.6% of participants and the low mortality rate of 0.5% are consistent with previous studies investigating the clinical outcomes of individuals with inflammatory bowel diseases (IBD). A study by Johnson et al. (13) reported a similar proportion of participants experiencing complications, while a population-based study by Thompson et al. (15) found a comparable low mortality rate among individuals with Crohn’s disease and ulcerative colitis (10, 14).

In terms of remission rates, our study found that 51.7% of participants achieved remission. This finding is consistent with a study by Brown et al. (11), which reported similar remission rates in a cohort of IBD patients undergoing a specific treatment protocol (15).

Overall, our study’s findings corroborate and extend existing research on the demographic characteristics, disease distribution, smoking habits, complications, mortality, and remission rates of individuals with Crohn’s disease and ulcerative colitis. However, further research is warranted to explore the underlying mechanisms behind these associations and to validate our findings in larger and more diverse populations.

The findings of this study contribute to the growing body of literature examining the association between smoking variables and complications in Crohn’s disease. The observed significant difference in complications among non-smokers, smokers, and secondary smokers aligns with previous research indicating that smoking is a risk factor for adverse outcomes in Crohn’s disease (6, 16). These findings suggest that both active smoking and exposure to secondhand smoke can increase the risk of complications in Crohn’s disease patients (6, 16).

In contrast, the lack of a significant association between the type of smoking and complications is consistent with some previous studies (17). This implies that the detrimental effects of smoking on Crohn’s disease a to active smoking but may also extend to passive smoking or other forms of exposure to tobacco smoke (17).

The trend observed between the number of cigarettes smoked per day and complications, although not statistically significant, is in line with other studies reporting a dose-response relationship between smoking intensity and adverse outcomes in Crohn’s disease (16, 17). Further investigations with larger sample sizes may be necessary to establish a significant association between smoking intensity and complications (16, 17).

Interestingly, the significant impact of smoking duration on complications contrasts with some previous studies reporting a positive association between longer smoking duration and worse outcomes in Crohn’s disease (16, 17). However, these findings are consistent with other studies that have suggested a potential protective effect of long-term smoking on the course of Crohn’s disease (16–18). The underlying mechanisms for this observed phenomenon remain unclear and require further investigation. This study highlights the complex relationship between smoking variables and Crohn’s disease complications. While smoking status and duration showed significant associations, type and daily number of cigarettes showed trends but did not reach statistical significance.

The findings of this investigation align with previous studies that have explored the association between smoking and complications of ulcerative colitis (UC). For instance, a study conducted by Smith et al. Observed a similar correlation between smoking status and UC complications, reporting complication rates of 50% in non-smokers, 25% in smokers, and 30% in secondary smokers. This consistency in results across different studies provides robust evidence of the impact of smoking on UC complications (19).

However, the borderline association observed between smoking duration and UC complications in this investigation is not consistent with the findings of a study conducted by Johnson et al. Johnson et al. reported a significant positive relationship between smoking duration and UC complications, with longer smoking durations being associated with higher complication rates (20). The discrepancy between these findings may be attributed to differences in sample characteristics, study design, or other contextual factors. Further research is needed to reconcile these contrasting results and identify the precise role of smoking duration in UC complications.

In addition, it is worth noting that while smoking type, smoking frequency per day, and a smoking duration of 6–10 years did not reach statistical significance in relation to UC complications in this investigation, other studies have reported varying results. For example, a study by Anderson et al. found a significant association between smoking frequency and UC complications, with higher daily smoking rates being linked to increased complication rates (21).

Overall, the findings of this investigation contribute to the existing body of literature on the relationship between smoking and UC complications. They corroborate previous research regarding smoking status but introduce some discrepancies concerning smoking duration and other smoking variables. Future studies should aim to replicate and expand upon these findings, considering a larger and more diverse sample population, to provide a comprehensive understanding of the complex relationship between smoking and UC complications.

Smoking’s protective effect against ulcerative colitis (UC) has been extensively studied, although the precise mechanisms remain incompletely understood. One proposed mechanism involves the immunomodulatory effects of nicotine, a key component of tobacco smoke. Nicotine has been shown to inhibit the production of pro-inflammatory cytokines such as tumor necrosis factor-alpha (TNF-α) and interleukin-1 beta (IL-1β) in the colonic mucosa, potentially dampening the inflammatory response implicated in UC development. Furthermore, nicotine has been found to reduce the expression of adhesion molecules on endothelial cells, thereby inhibiting the migration of leukocytes into inflamed tissues, a hallmark of UC pathology. Additionally, nicotine has been shown to alter the composition of the gut microbiota, promoting the growth of beneficial bacteria and suppressing harmful species associated with UC pathogenesis. While smoking’s protective effects against UC are evident in epidemiological studies, its overall health risks necessitate caution and further research into safer alternatives for UC prevention and management (22).

The findings from this study align with previous research that has explored the association between smoking and remission rates in ulcerative colitis. Several studies have reported a higher likelihood of remission among smokers compared to non-smokers. For instance, a study by Cosnes et al. (16) found that current smoking was associated with a significantly higher remission rate in ulcerative colitis patients. Similarly, Mahid et al. (6) conducted a meta-analysis of multiple studies and reported that smoking was associated with a higher odds of remission in ulcerative colitis (6, 16).

However, it is important to note that the impact of smoking on ulcerative colitis remission is still a topic of debate. While some studies have observed a positive association, others have reported conflicting or even negative findings. For example, a study by Calkins et al. (23) found that smoking was associated with an increased risk of disease relapse in ulcerative colitis patients (23). Additionally, a systematic review by Singh et al. (24) reported inconsistent results across various studies, highlighting the need for further research to clarify the relationship between smoking and remission outcomes in ulcerative colitis (23). The study’s limitations include not showing significant associations between remission rates and smoking type, frequency, or duration. Future research should explore these variables’ impact on remission outcomes in ulcerative colitis.

In conclusion, the findings from this study add to the existing body of literature on the association between smoking and remission rates in ulcerative colitis. While the results align with previous research suggesting that smokers may have higher remission rates, the topic remains complex and controversial. Further investigations, considering variables such as smoking type, frequency, and duration, are needed to provide a comprehensive understanding of the intricate relationship between smoking and remission outcomes in ulcerative colitis.

However, it is important to note that the impact of smoking on ulcerative colitis remission is still a topic of debate. While some studies have observed a positive association, others have reported conflicting or even negative findings. For example, a study by Calkins et al. (23) found that smoking was associated with an increased risk of disease relapse in ulcerative colitis patients. Additionally, a systematic review by Singh et al. (24) reported inconsistent results across various studies, highlighting the need for further research to clarify the relationship between smoking and remission outcomes in ulcerative colitis (24).

In light of these contrasting findings, it is crucial to consider the limitations of the current study. The presented data did not demonstrate significant associations between remission rates and smoking type, frequency, or duration. However, it is worth noting that these variables have been investigated in other studies, and their impact on remission outcomes has been a subject of ongoing research. Future studies should take into account these variables and explore their potential influence on remission rates in ulcerative colitis.

Regarding the correlation between Crohn’s and remission, it showed a nonsignificant correlation. However, a study by Sangmin Lee showed that smoking can affect certain anti-TNF drugs like infliximab or adalimumab. Therefore, we can ask researchers in the future to see the effect of smoking on Crohn’s patients with different drugs to have a clear correlation (25).

## Conclusion

Our study showed that smoking is inversely correlated with UC and having complications; furthermore, smoking patients with UC seem to have a higher remission rate. However, smoking in Crohn’s has more complications.

### Limitation

Since our study was retrospective, it had all the drawbacks and restrictions associated with such research. Since all patients were included in this single-center study, it is not possible to extrapolate the findings to a wider population. We recommend having more studies focused on the type of smoking and the relationship between smoking and the type of medication used in IBD patients.

## Data availability statement

The raw data supporting the conclusions of this article will be made available by the authors, without undue reservation.

## Ethics statement

The studies involving humans were approved by King Abdullah International Medical Research Center (KAIMRC) (NRJ23J/253/09). The studies were conducted in accordance with the local legislation and institutional requirements. Written informed consent for participation was not required from the participants or the participants’ legal guardians/next of kin in accordance with the national legislation and institutional requirements.

## Author contributions

BA: Data curation, Methodology, Writing – original draft, Writing – review & editing. JA: Conceptualization, Data curation, Investigation, Software, Writing – original draft, Writing – review & editing. ZB: Data curation, Formal analysis, Resources, Visualization, Writing – original draft, Writing – review & editing. RA: Conceptualization, Data curation, Investigation, Methodology, Writing – original draft, Writing – review & editing. FA: Conceptualization, Investigation, Methodology, Supervision, Validation, Writing – original draft, Writing – review & editing. MA: Formal analysis, Investigation, Methodology, Validation, Writing – original draft, Writing – review & editing.
